# Supplementary material for: CRISPR-Cas12a REC2 – NUC interactions drive target-strand cleavage and constrain trans cleavage
Source: bioRxiv. 2025 Mar 25:2025.03.23.644851. Preprint. [Version 1] doi: 10.1101/2025.03.23.644851 (PMC11974684; doi:10.1101/2025.03.23.644851)
Supplement: Supplement 1 [file media-1.pdf]

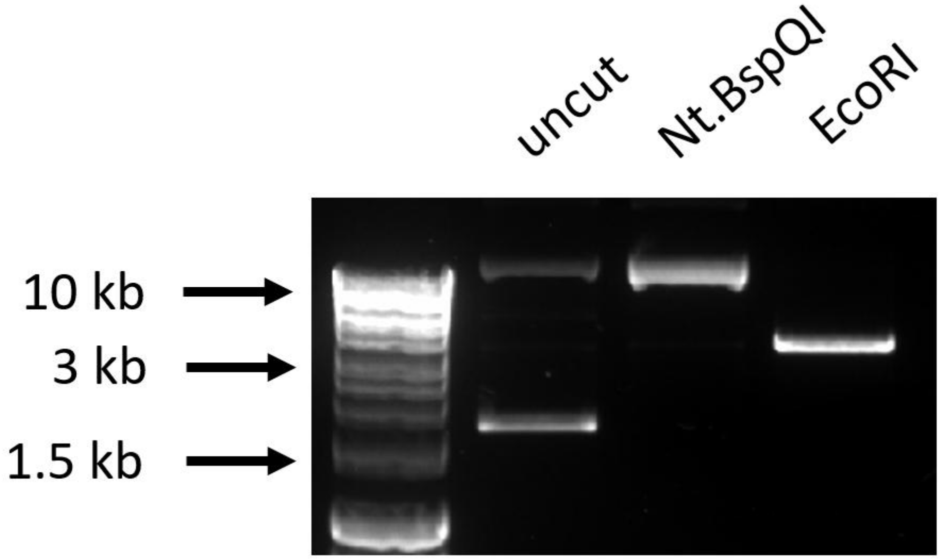

Figure S1: *Changes in target plasmid topology with nicking by Nt.BspQI, and linearisation with EcoRI.*

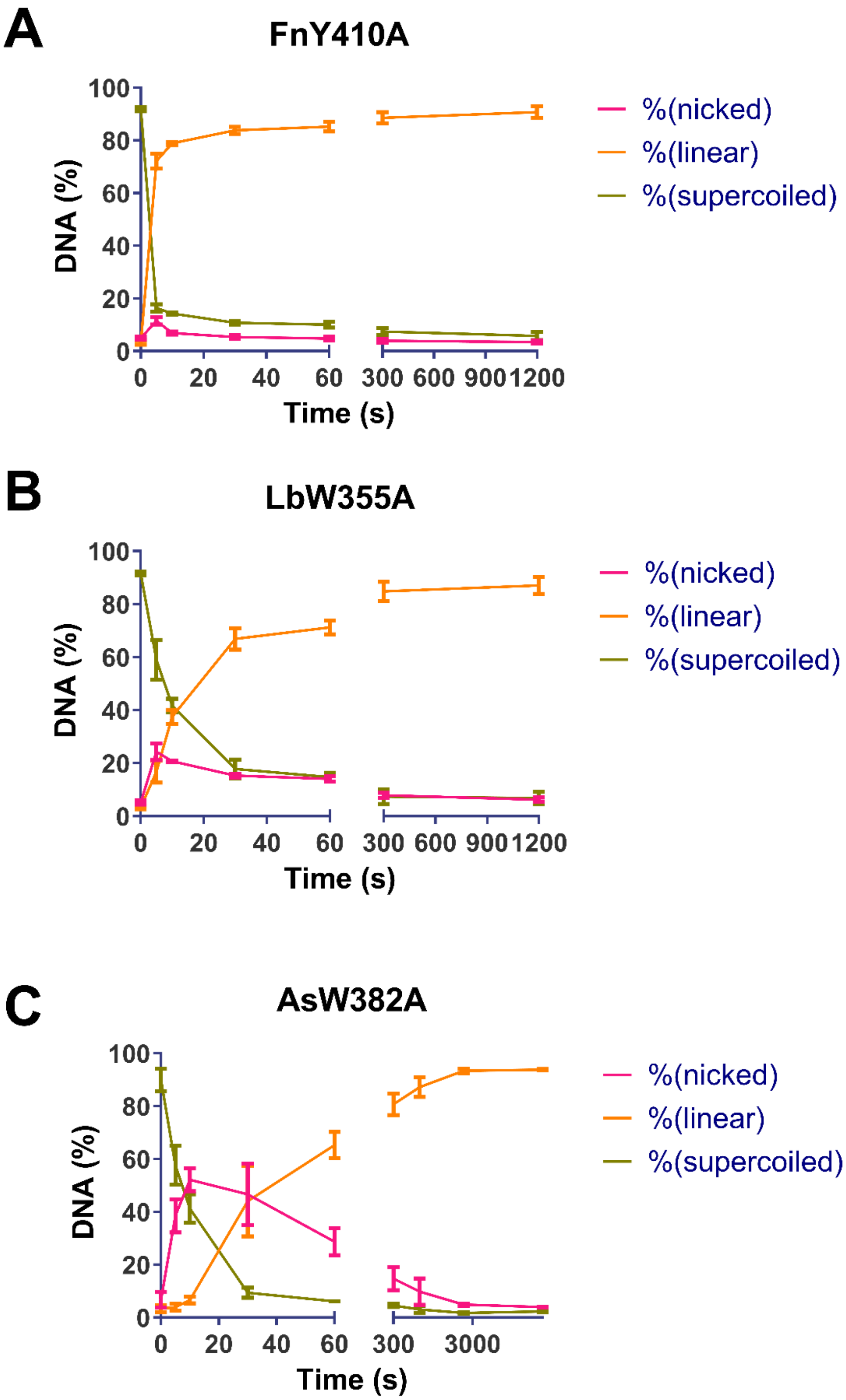

Figure S2: *Quantification of DNA fractions over time, with Cas12a mutant indicated. Line shows mean, error bars s.d.*

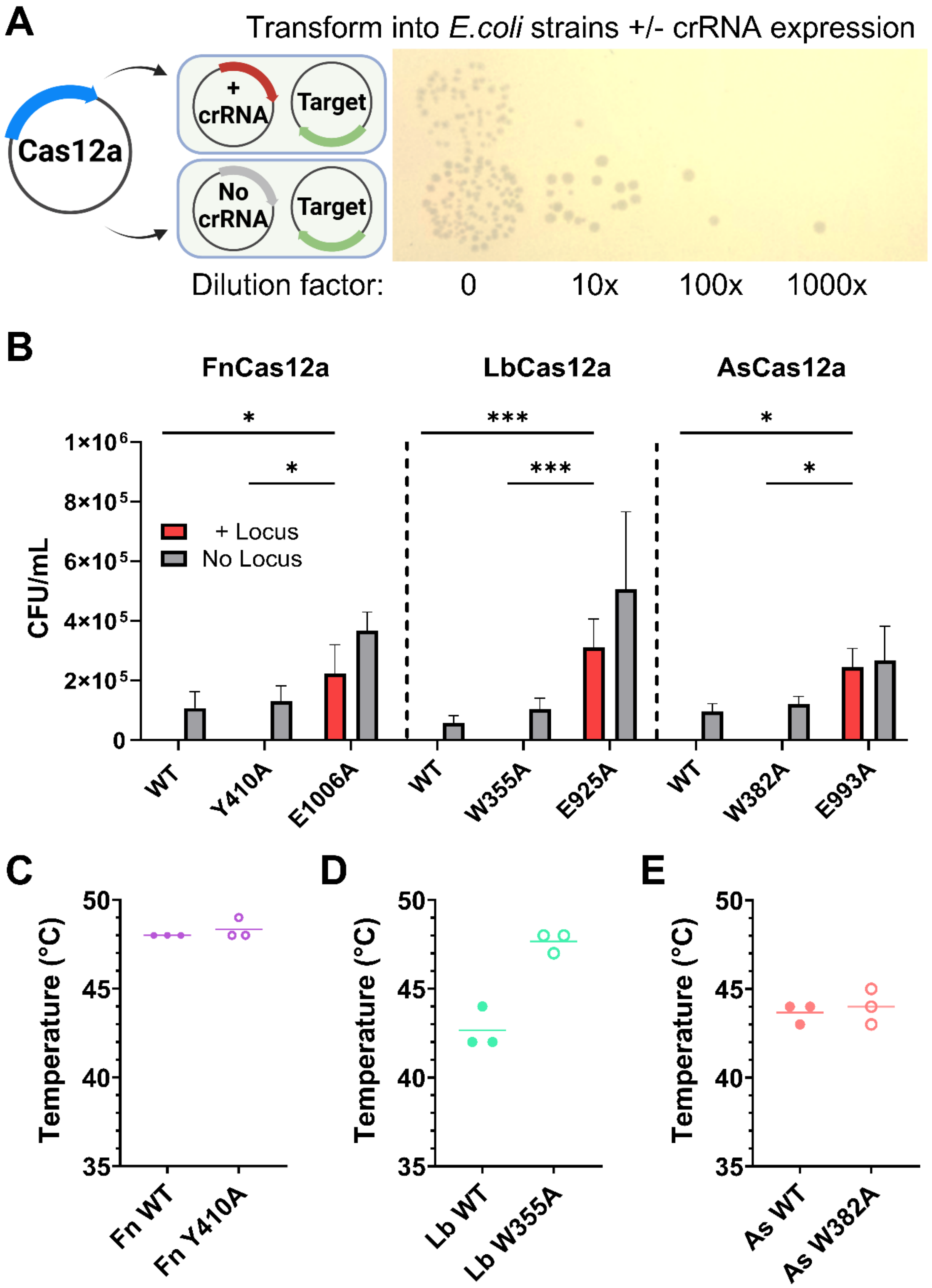

Figure S3: *(A) Outline of plasmid interference assay, image shows example transformation. (B) Mean colony forming units per mL (error bars show s.d.), for +/- crRNA conditions, Cas12a as indicated. Statistical significance evaluated by two-way ANOVA with Tukey's multiple comparison test (*p<0.1, **p<0.01, ***p<0.001). (C) Thermostability assay, melting point defined as fluorescence peak, line shows mean of three replicates.*

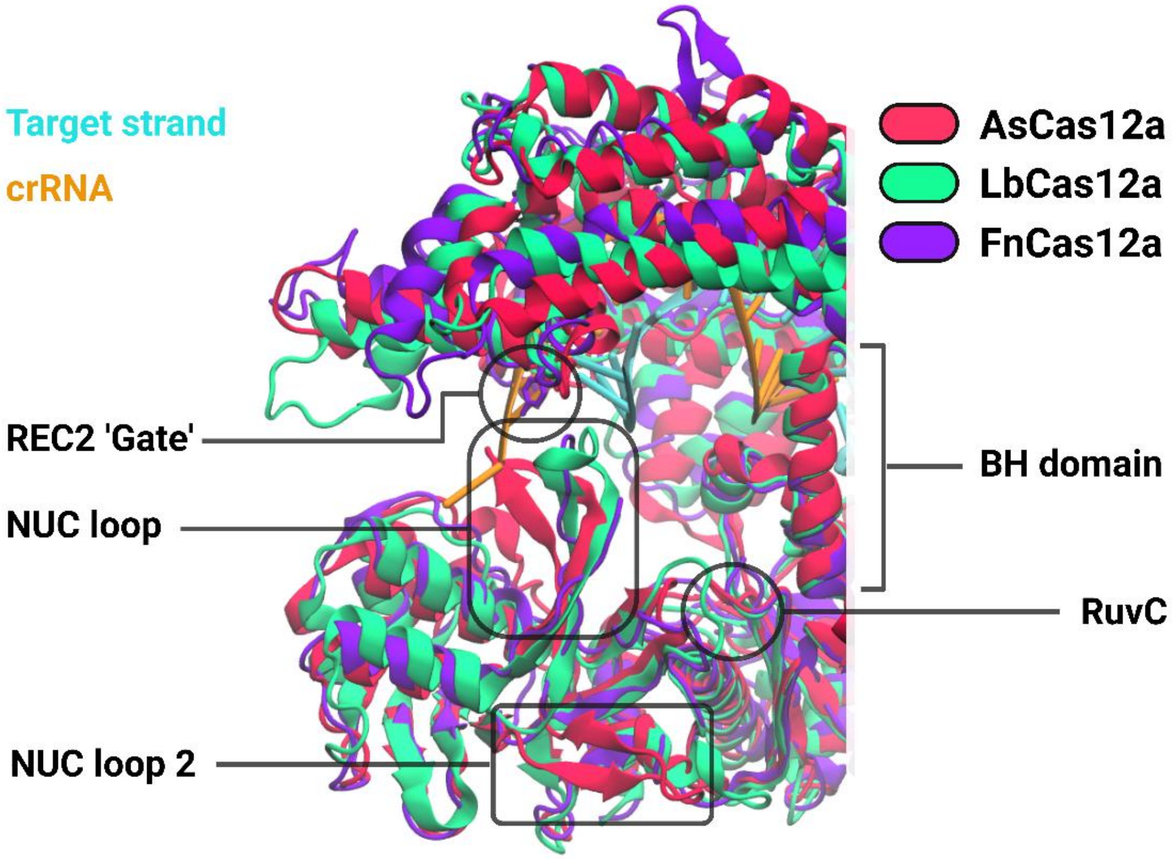

Figure S4: *Structural alignment of Cas12a orthologues, highlighting key features. AsCas12a (AF2 model, red), LbCas12a (AF2 model, bright teal), and FnCas12a (6GTG, purple).*

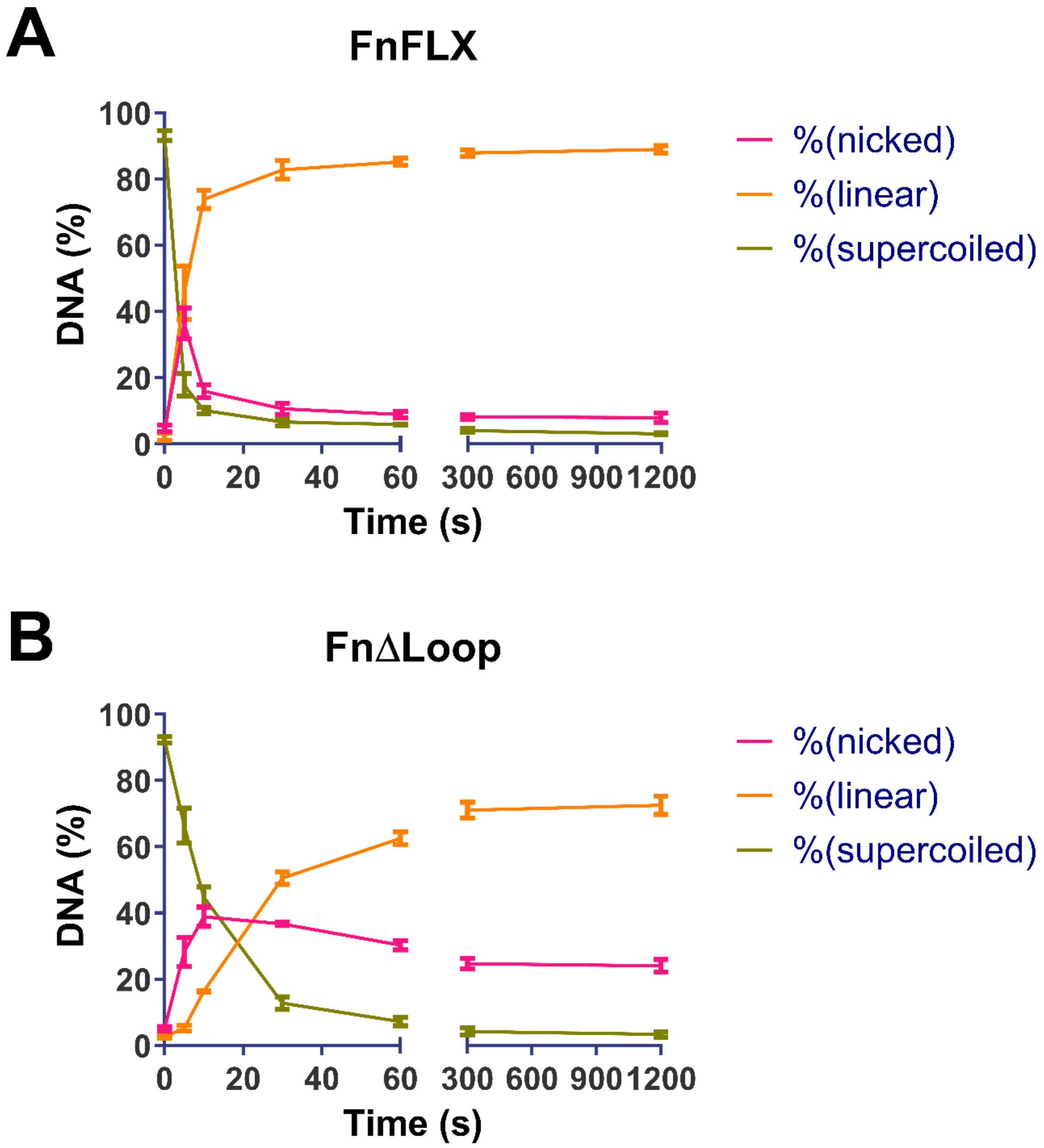

Figure S5: *Quantification of DNA fractions over time, with Cas12a mutant indicated. Line shows mean, error bars s.d.*

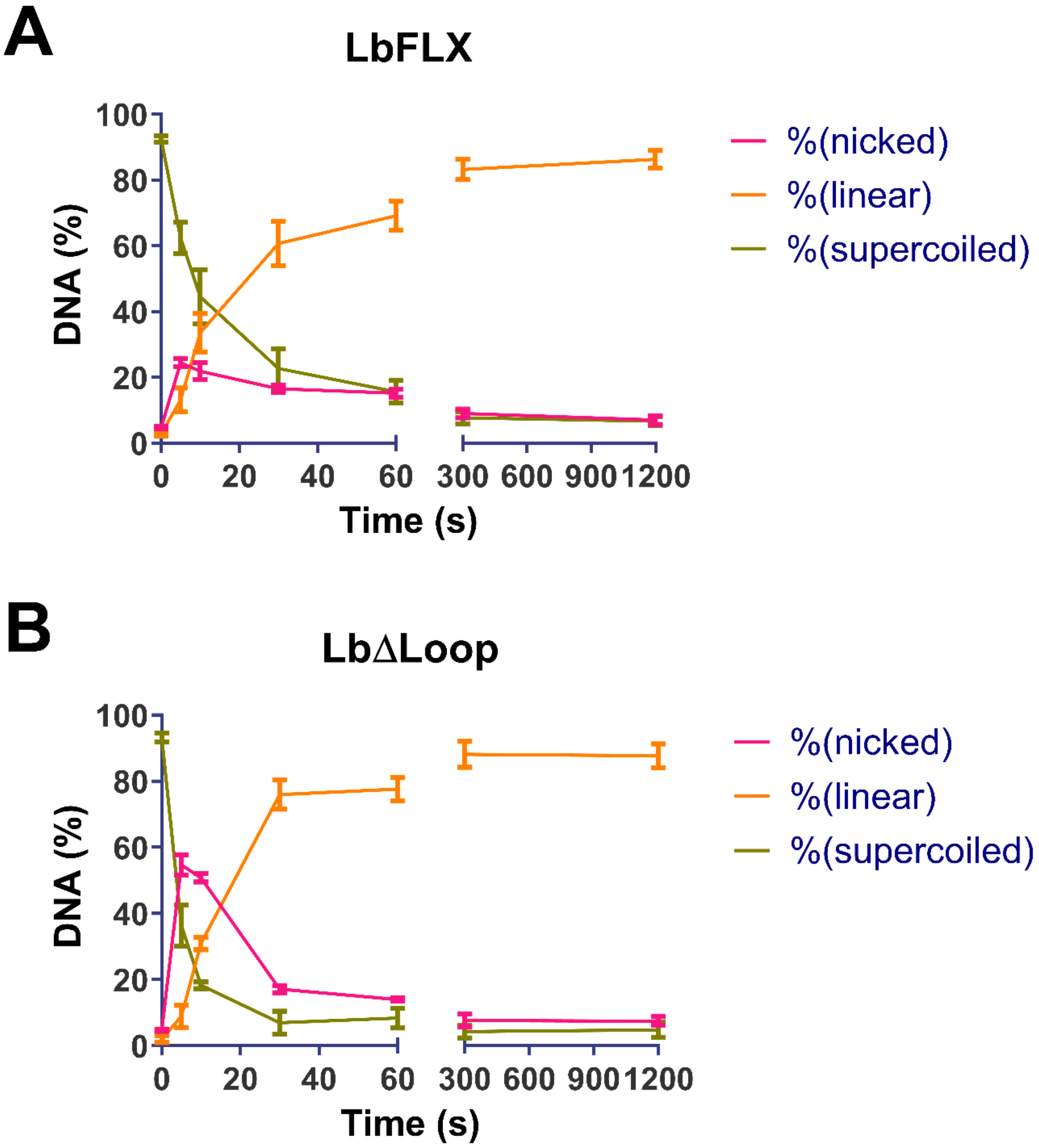

Figure S6: *Quantification of DNA fractions over time, with Cas12a mutant indicated. Line shows mean, error bars s.d.*

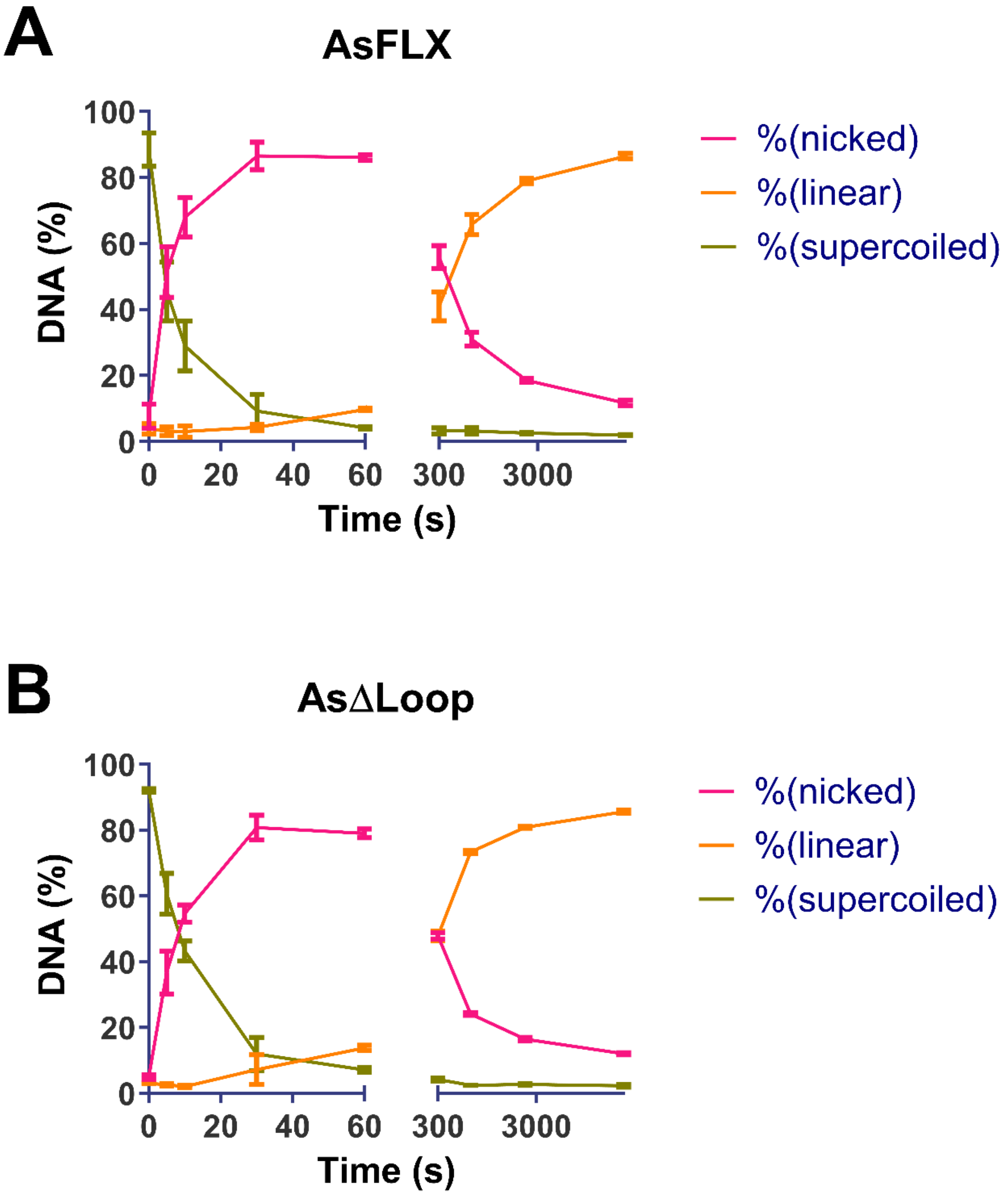

Figure S7: *Quantification of DNA fractions over time, with Cas12a mutant indicated. Line shows mean, error bars s.d.*

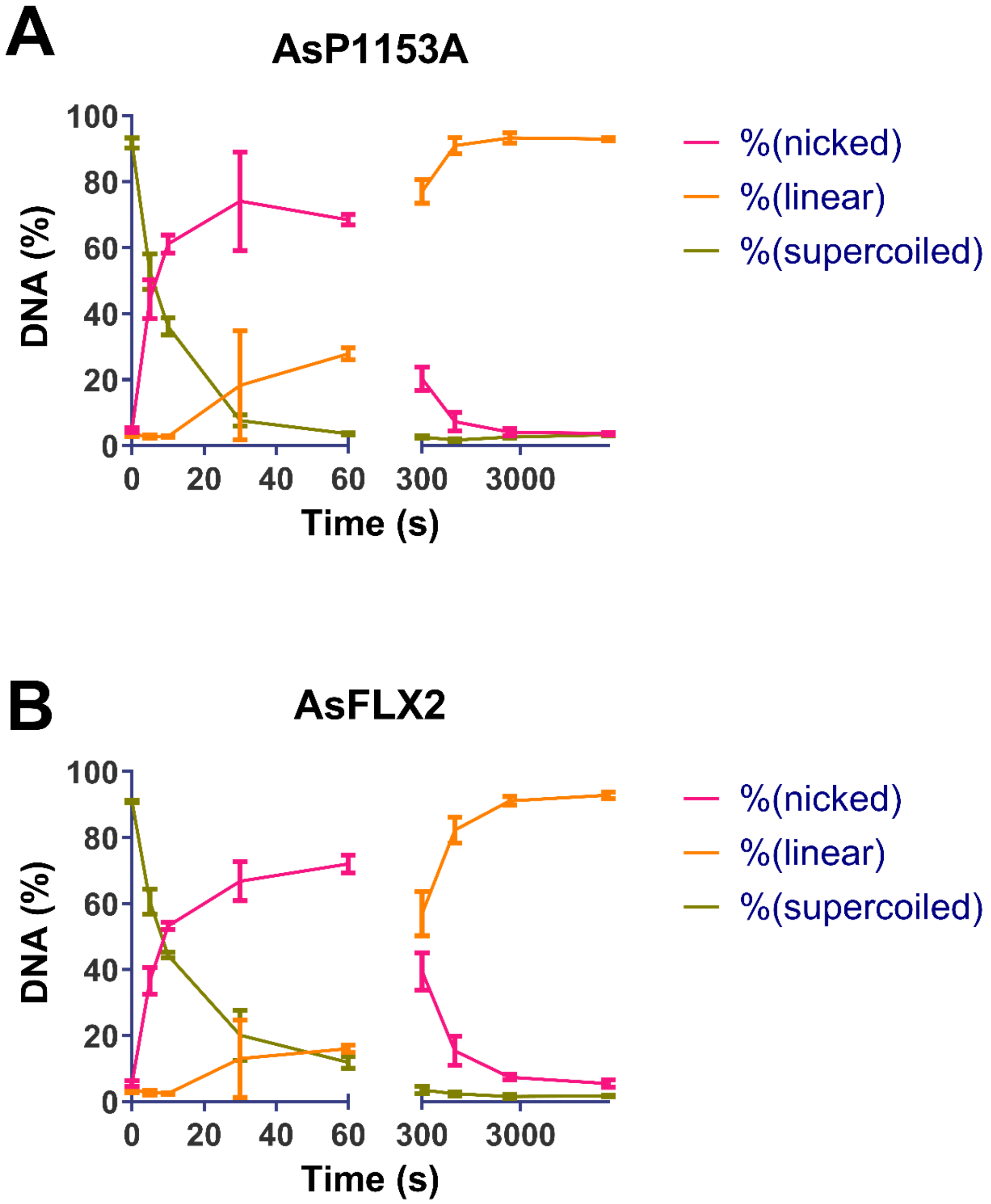

Figure S8: *Quantification of DNA fractions over time, with Cas12a mutant indicated. Line shows mean, error bars s.d.*

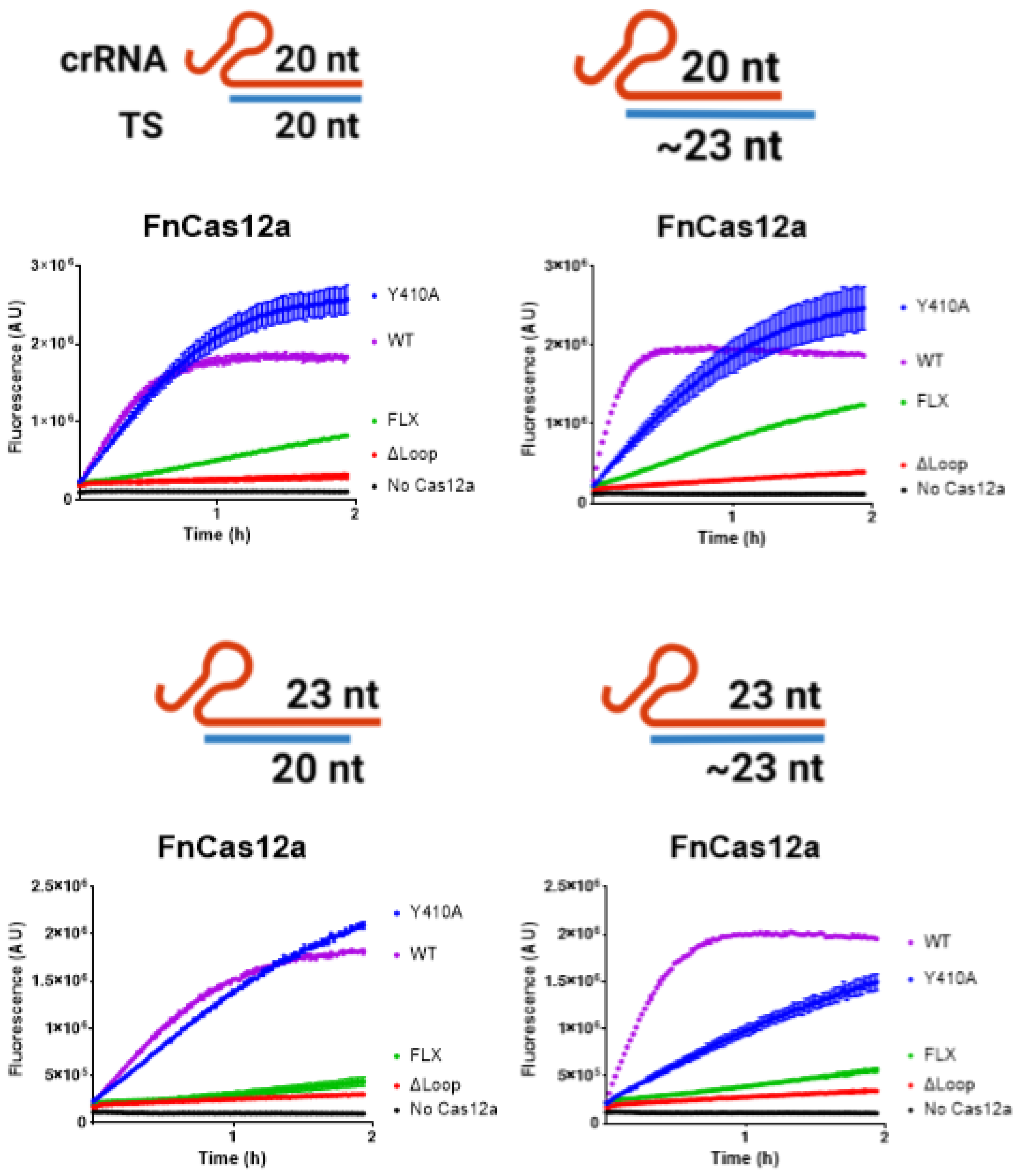

Figure S9: *Trans cleavage curves, with crRNA and TS combination indicated. Points show mean, error bars s.d.*

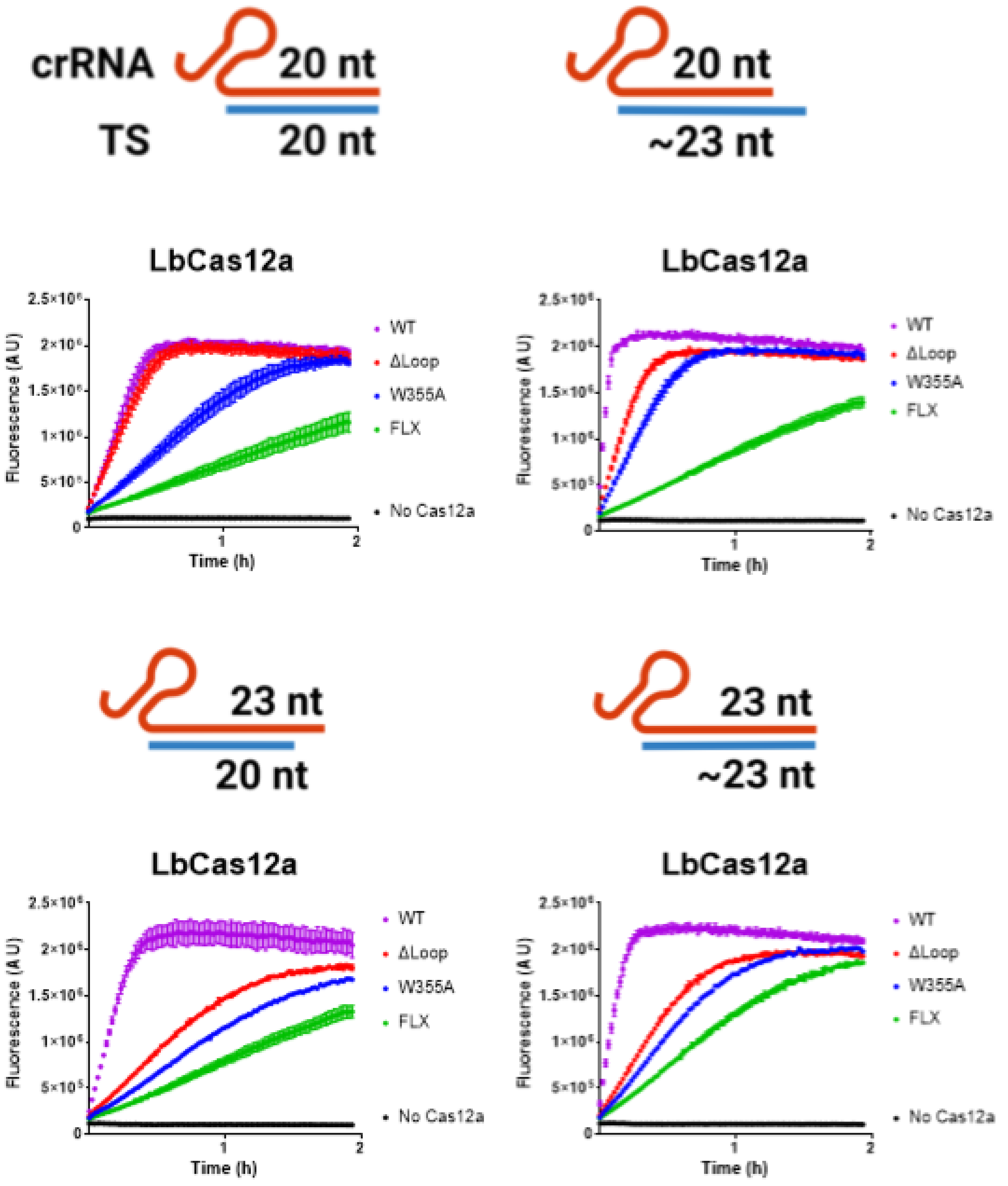

Figure S10: *Trans cleavage curves, with crRNA and TS combination indicated. Points show mean, error bars s.d.*

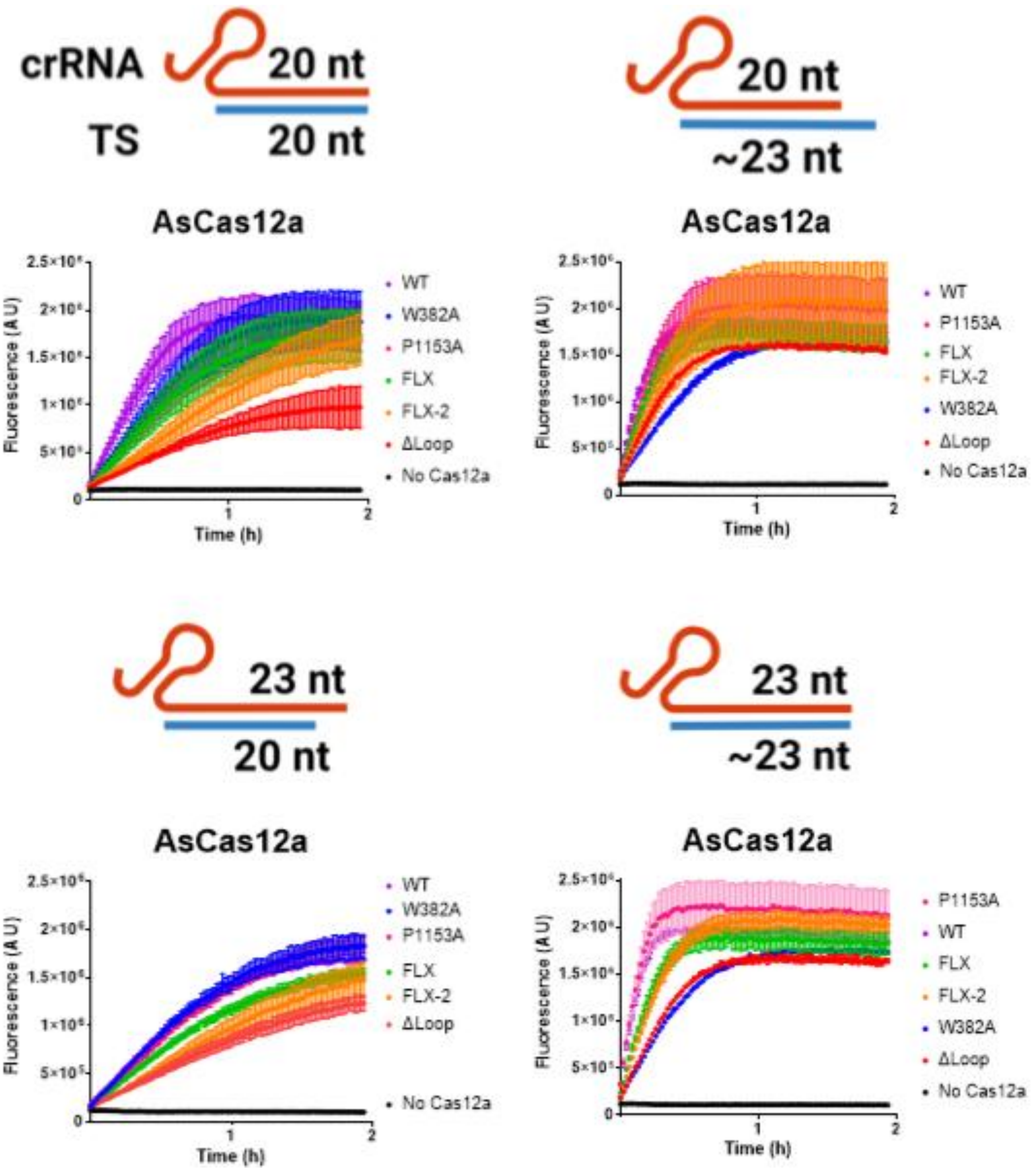

Figure S11: *Trans cleavage curves, with crRNA and TS combination indicated. Points show mean, error bars s.d.*

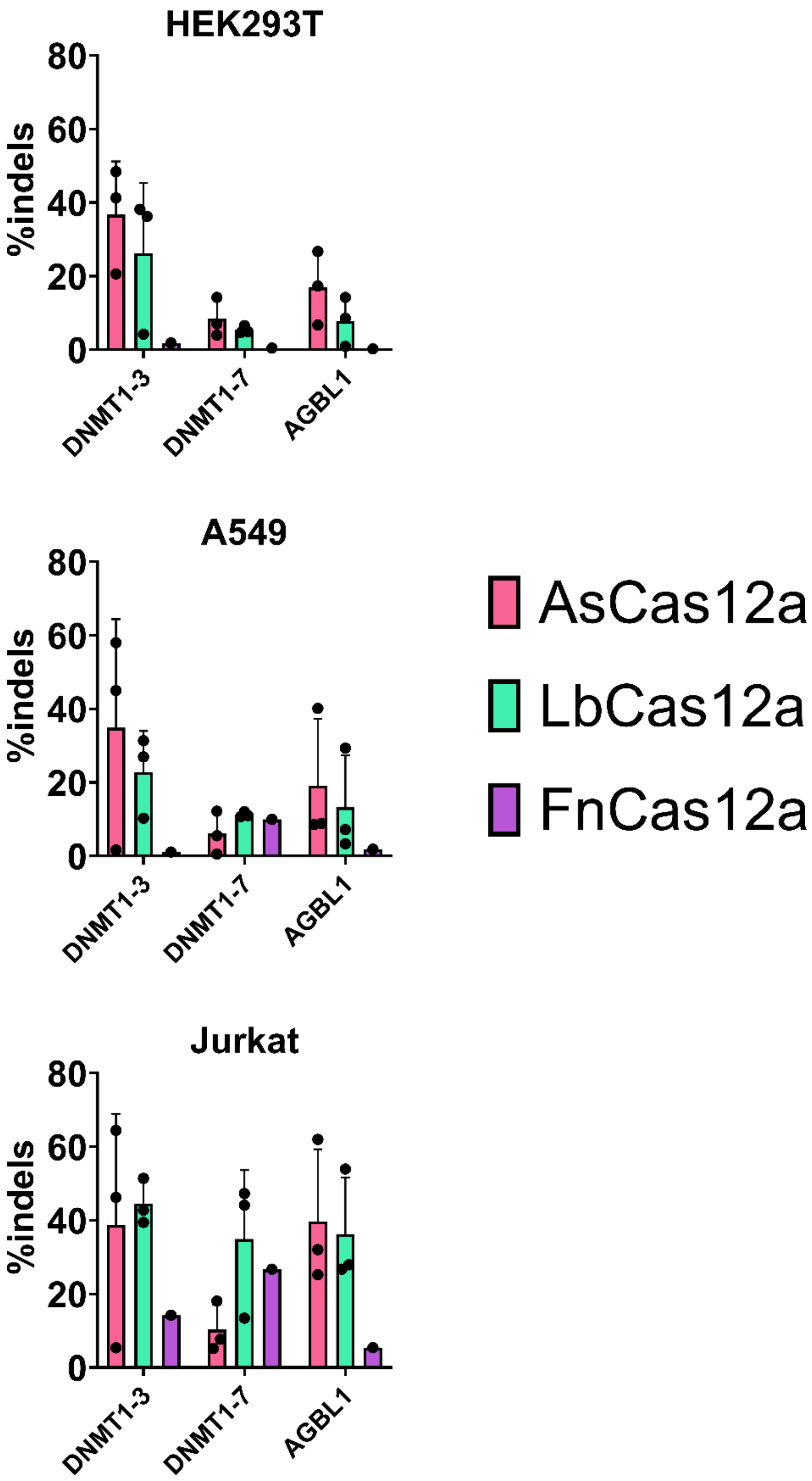

Figure S12: *Gene editing in cell line indicated, with crRNA indicated. Dots shown individual values, bar shows mean, error bars s.d. Note, n = 1 transformations performed for FnCas12a.*

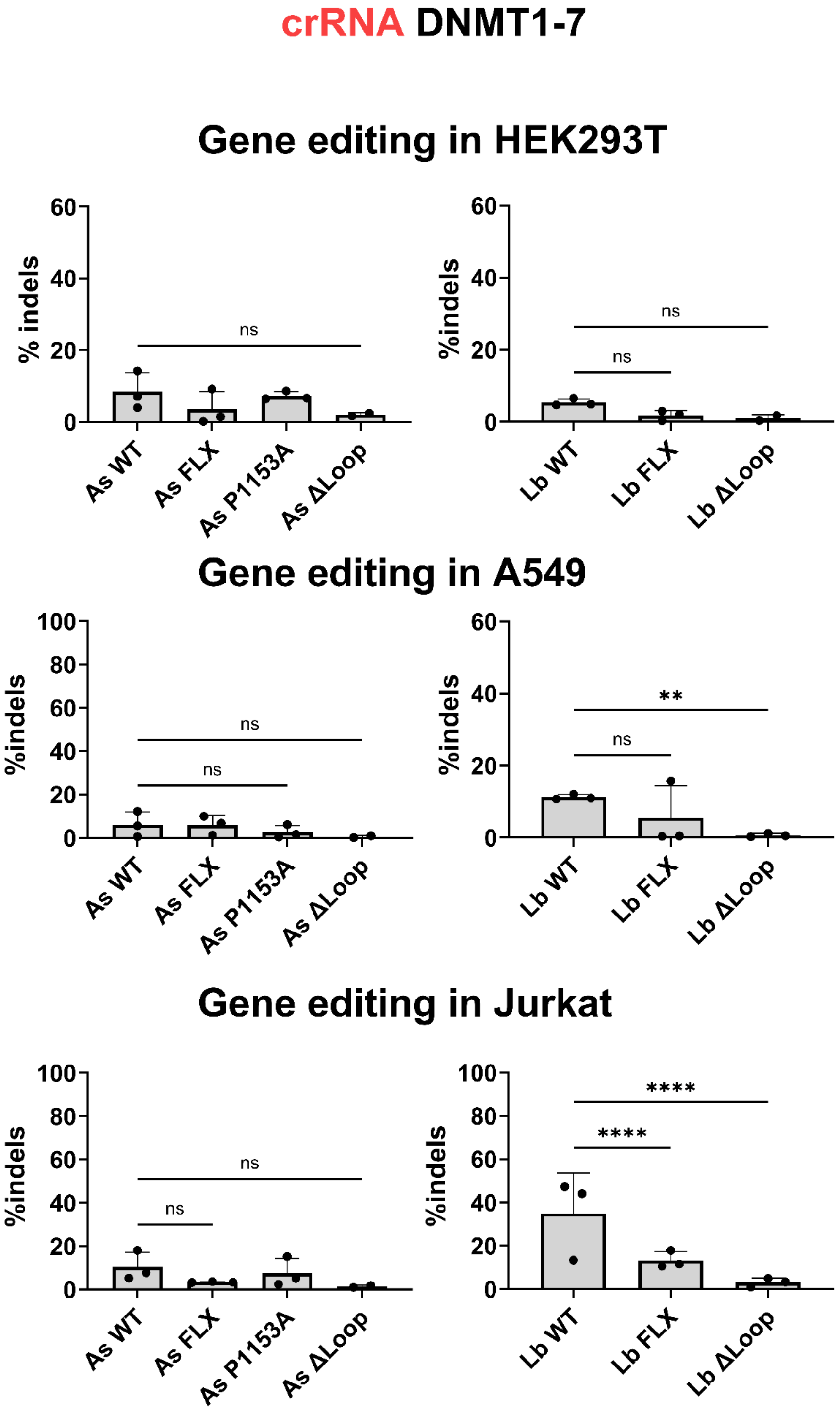

Figure S13: *Gene editing in cell line indicated, with crRNA DNMT1-7. Bars show mean, error bars s.d. Statistical significance evaluated by two-way ANOVA with Tukey's multiple comparison test (*p<0.1, **p<0.01, ***p<0.001, ****p<0.0001).*

crRNA DNMT1-3

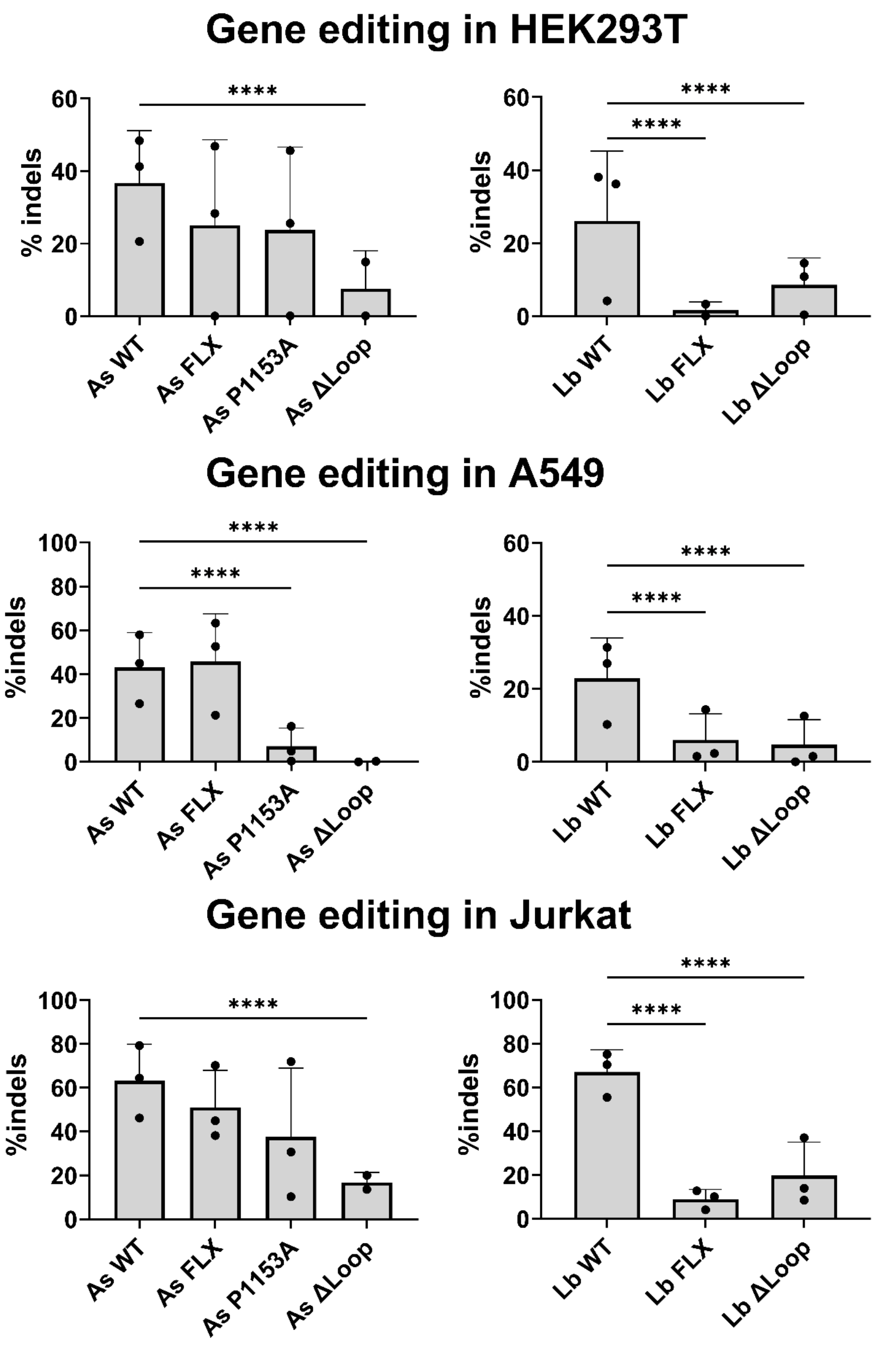

Figure S14: *Gene editing in cell line indicated, with crRNA DNMT1-3. Bars show mean, error bars s.d. Statistical significance evaluated by two-way ANOVA with Tukey's multiple comparison test (*p<0.1, **p<0.01, ***p<0.001, ****p<0.0001).*

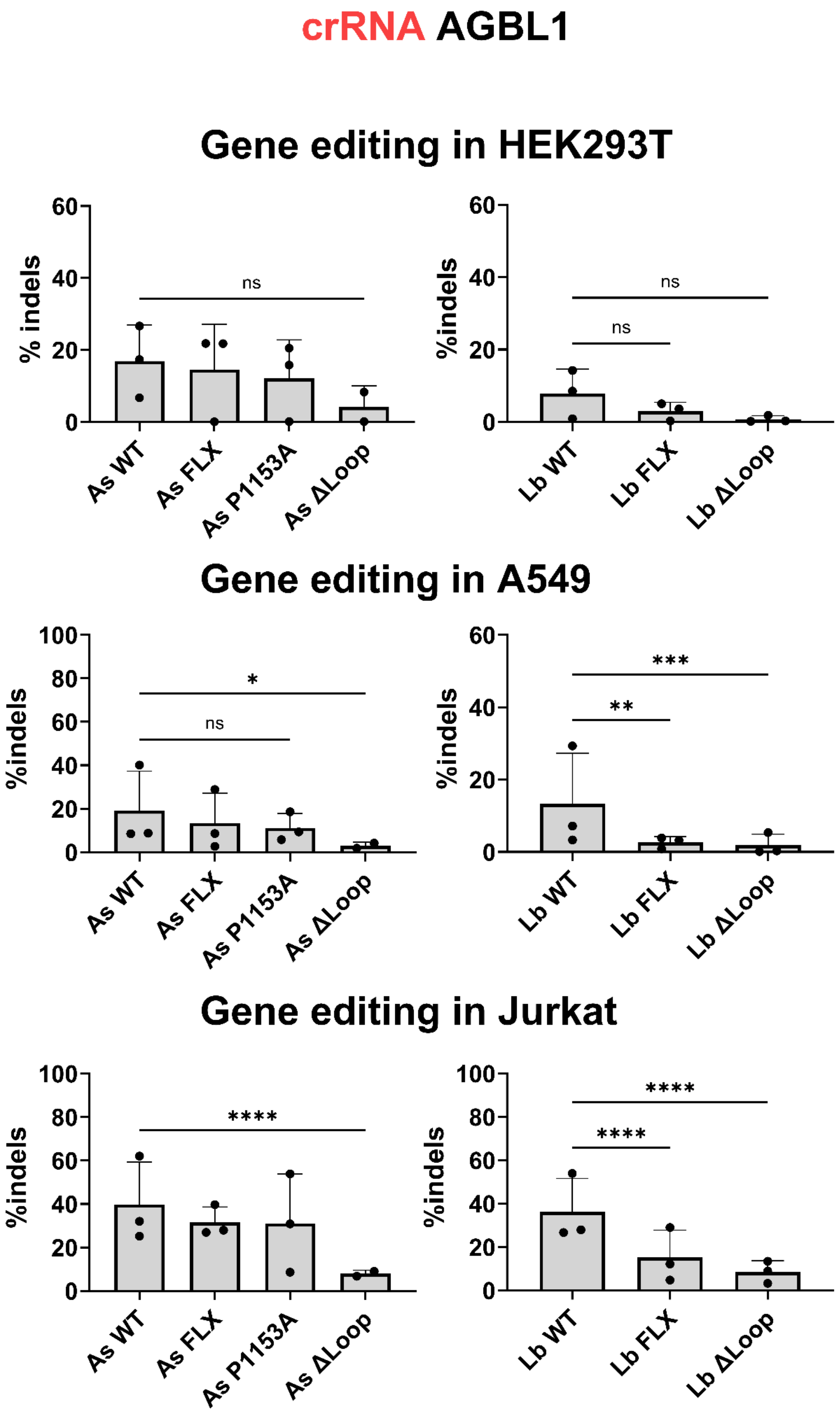

Figure S15: *Gene editing in cell line indicated, with crRNA AGBL1. Bars show mean, error bars s.d. Statistical significance evaluated by two-way ANOVA with Tukey's multiple comparison test (*p<0.1, **p<0.01, ***p<0.001, ****p<0.0001).*

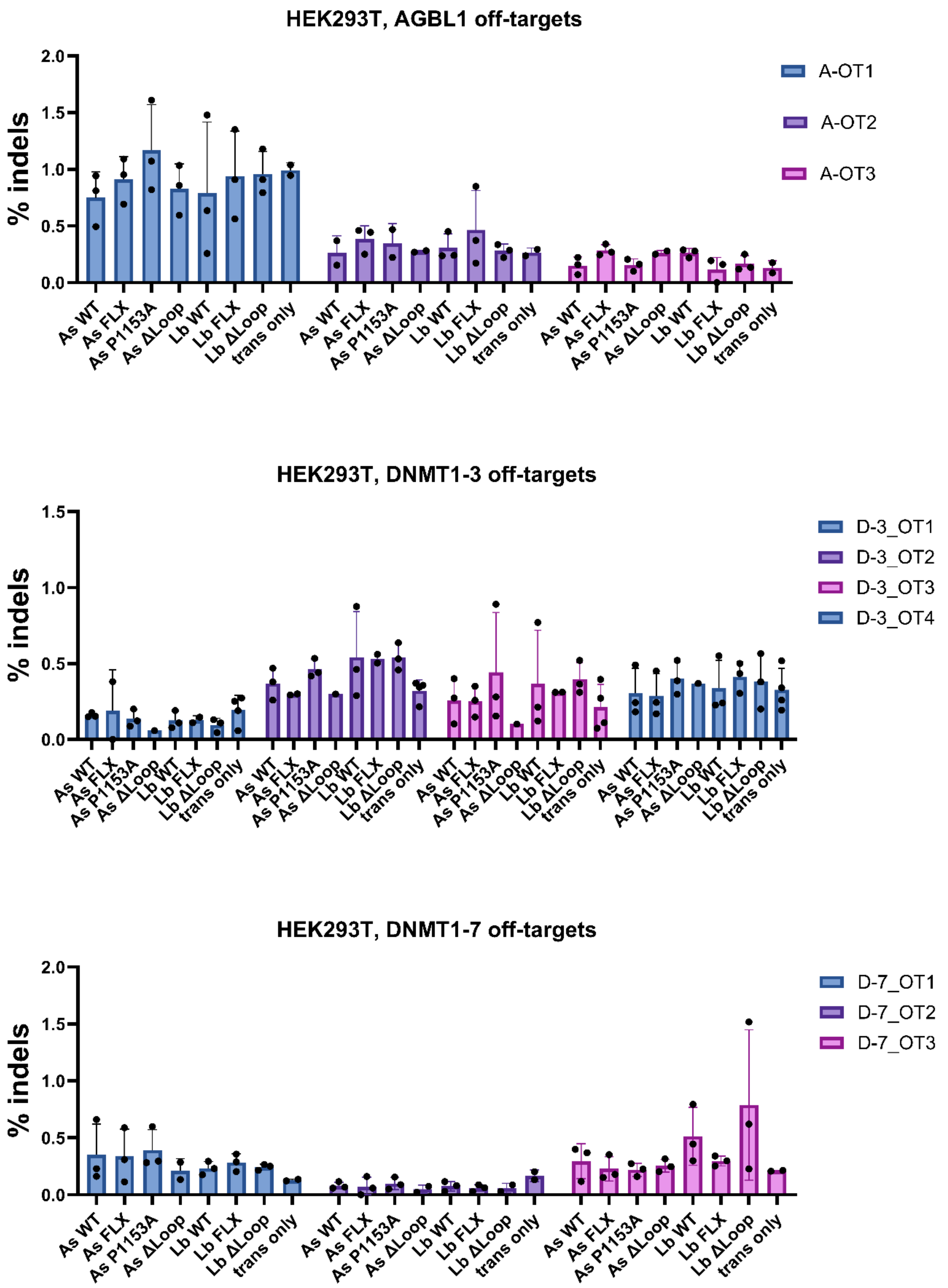

Figure S16: *Off-target (OT) editing with crRNA indicated, in HEK293T cell line. Bars shown mean, error bars s.d.*

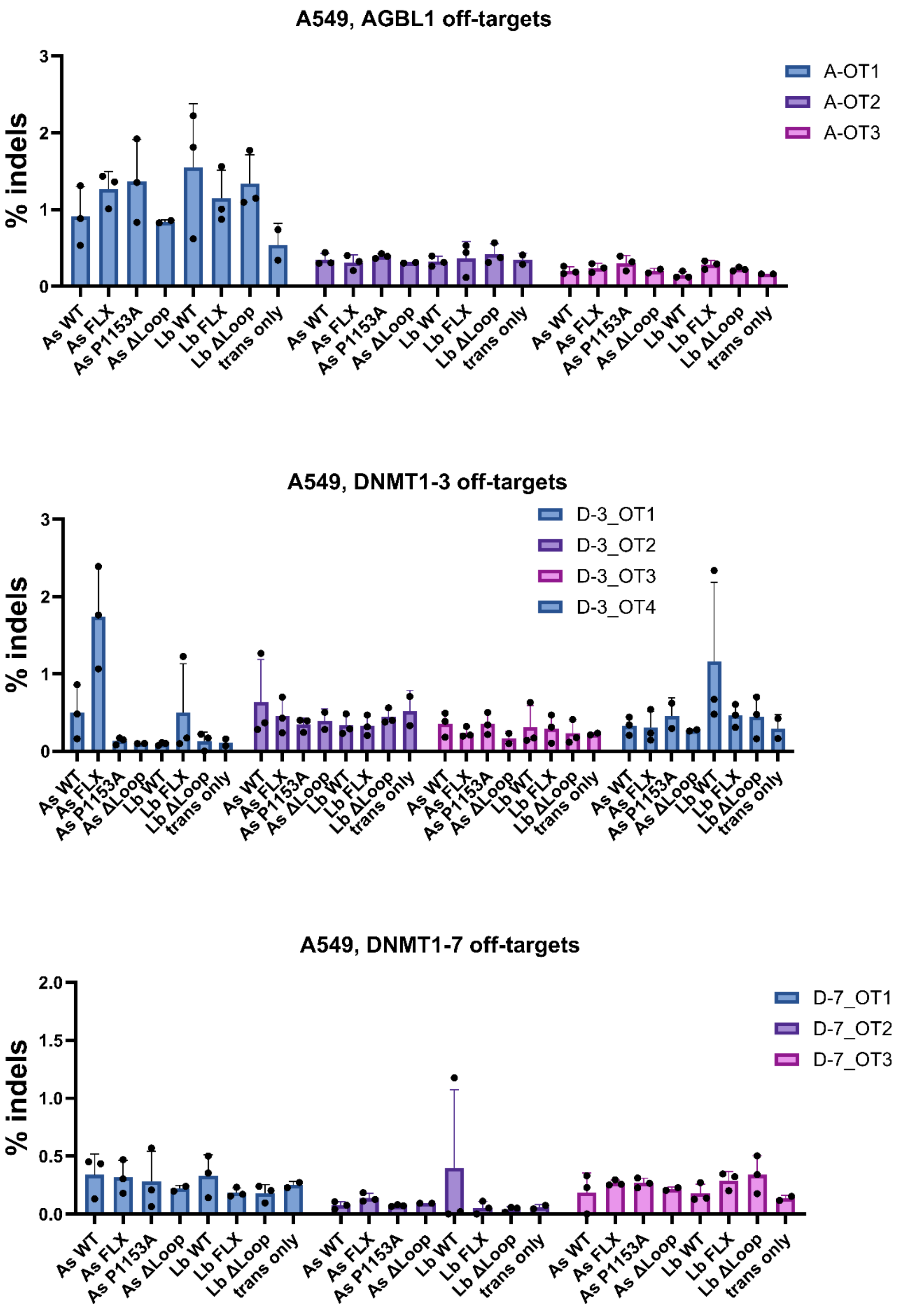

Figure S17: *Off-target (OT) editing with crRNA indicated, in A549 cell line. Bars shown mean, error bars s.d.*

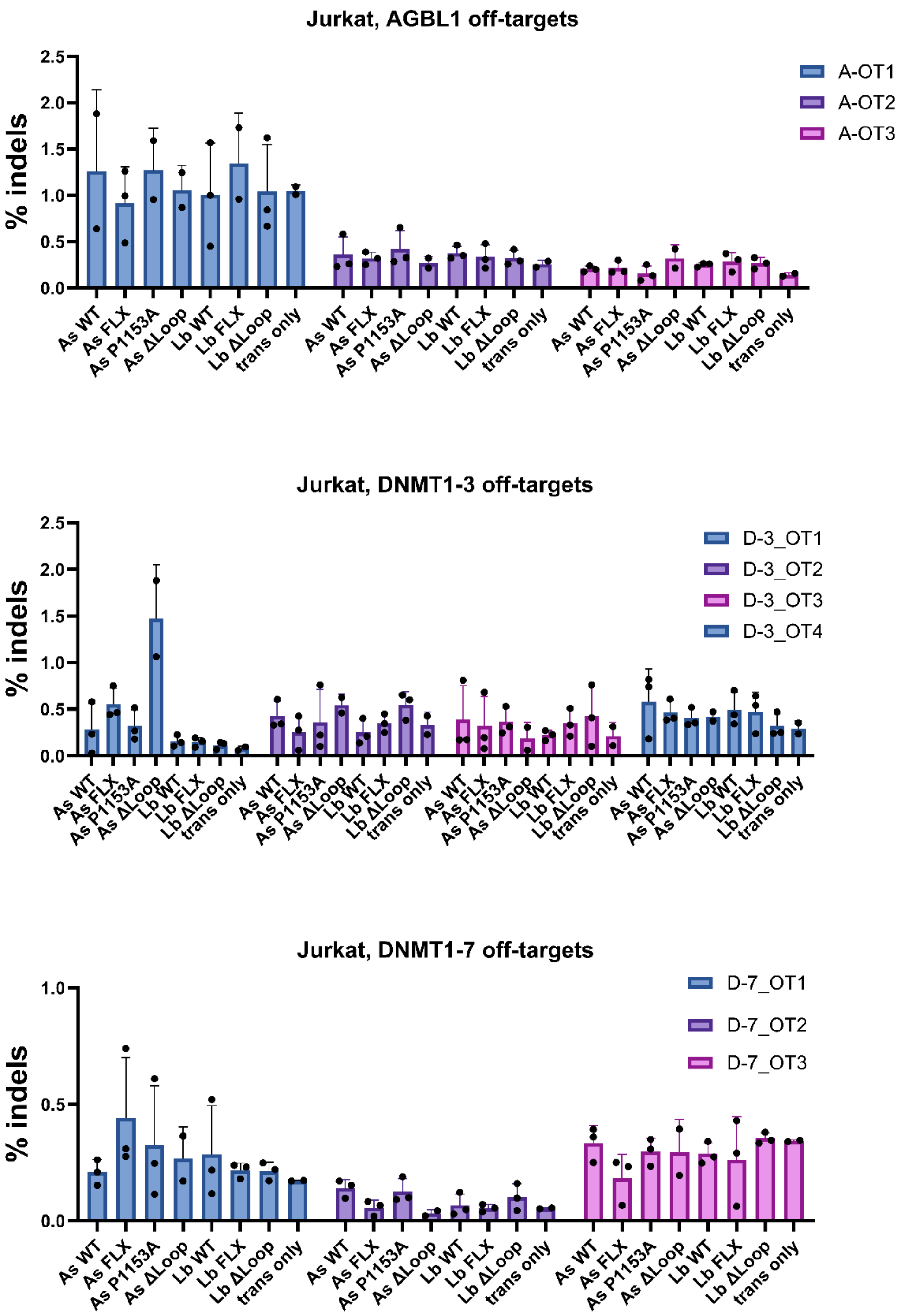

Figure S18: *Off-target (OT) editing with crRNA indicated, in Jurkat cell line. Bars shown mean, error bars s.d.*

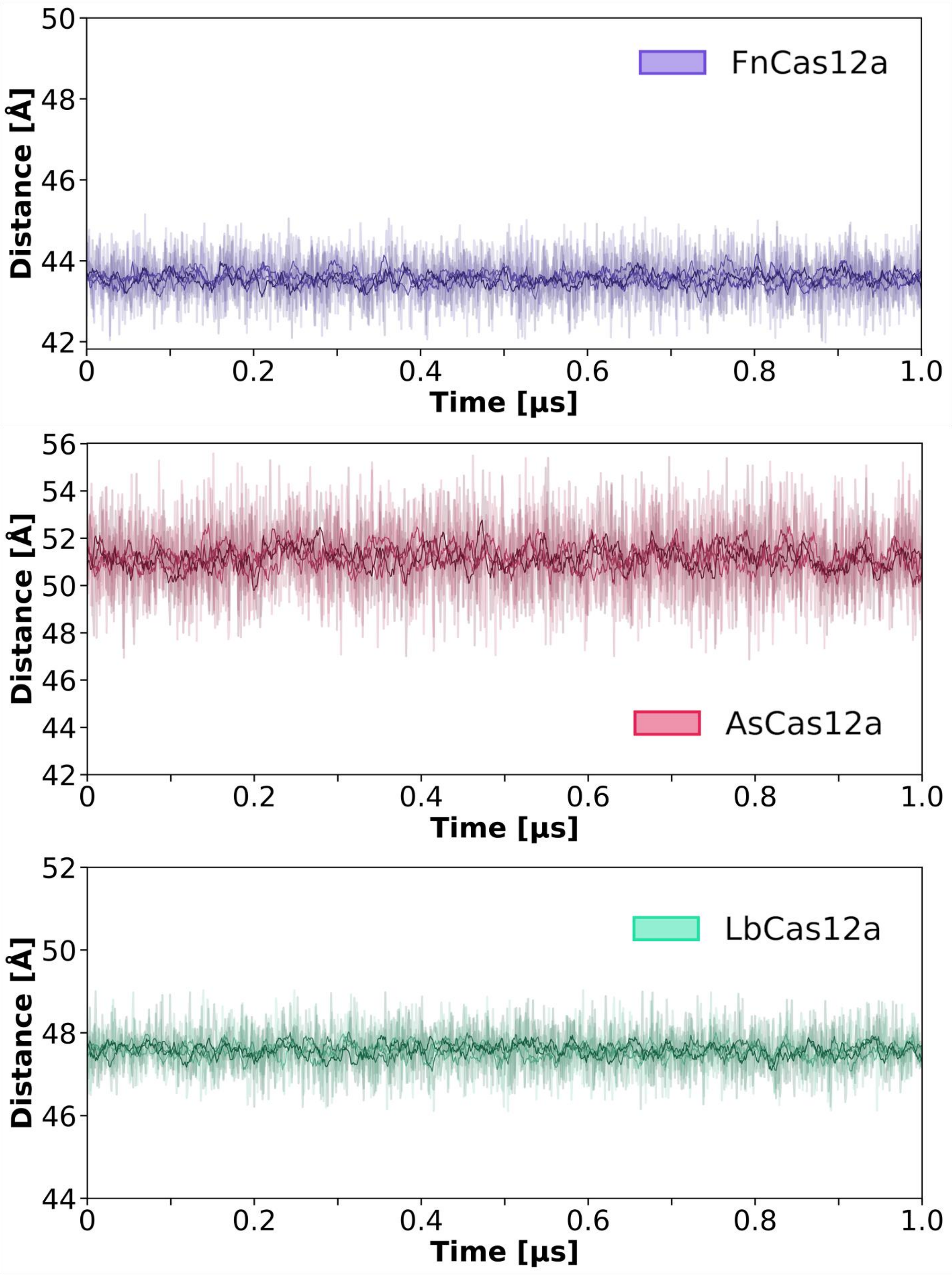

Figure S19: *Distance analysis of replicates of μs – length simulations.*

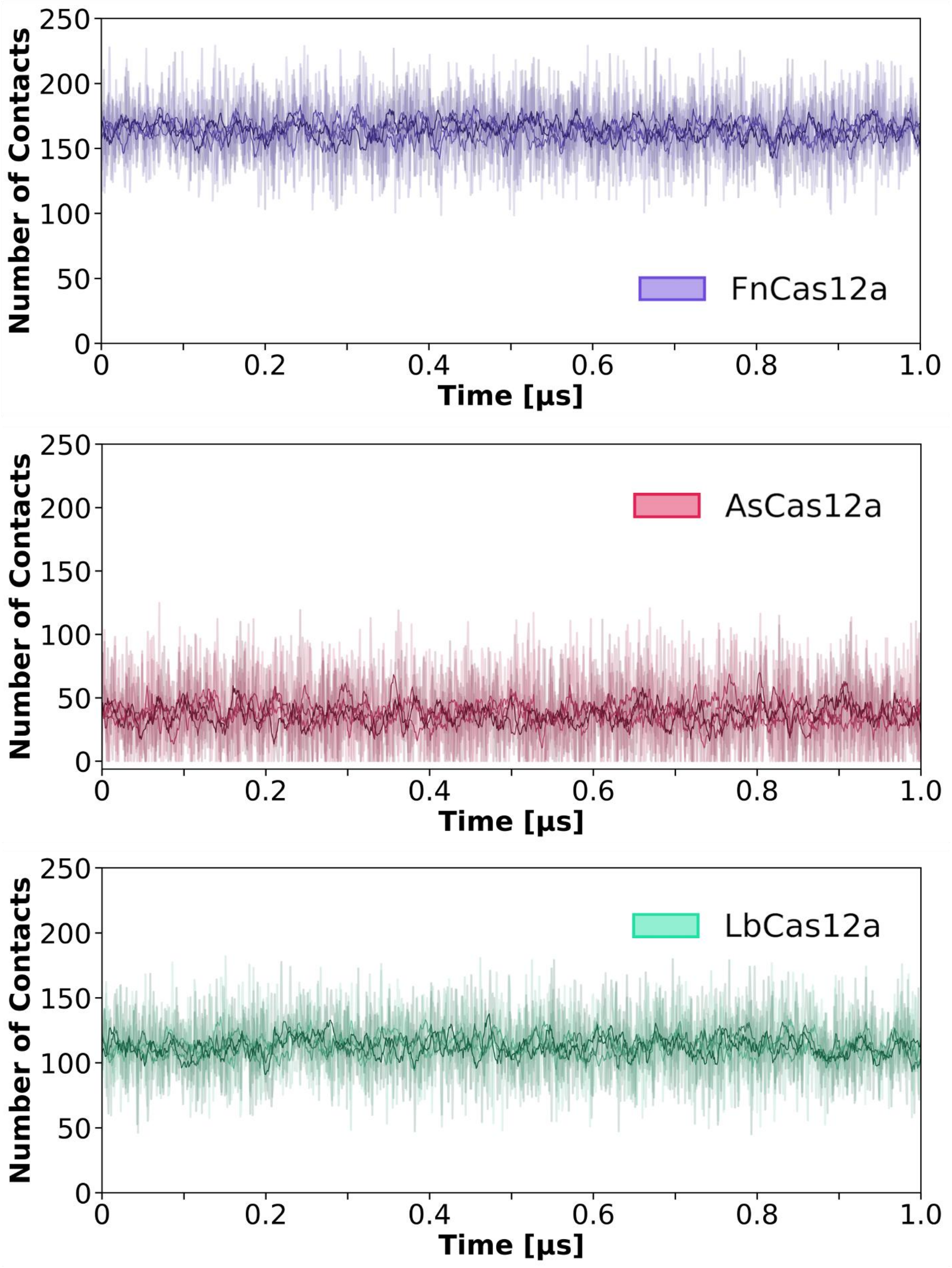

Figure S20: *Contact analysis of replicates of µs – length simulations.*

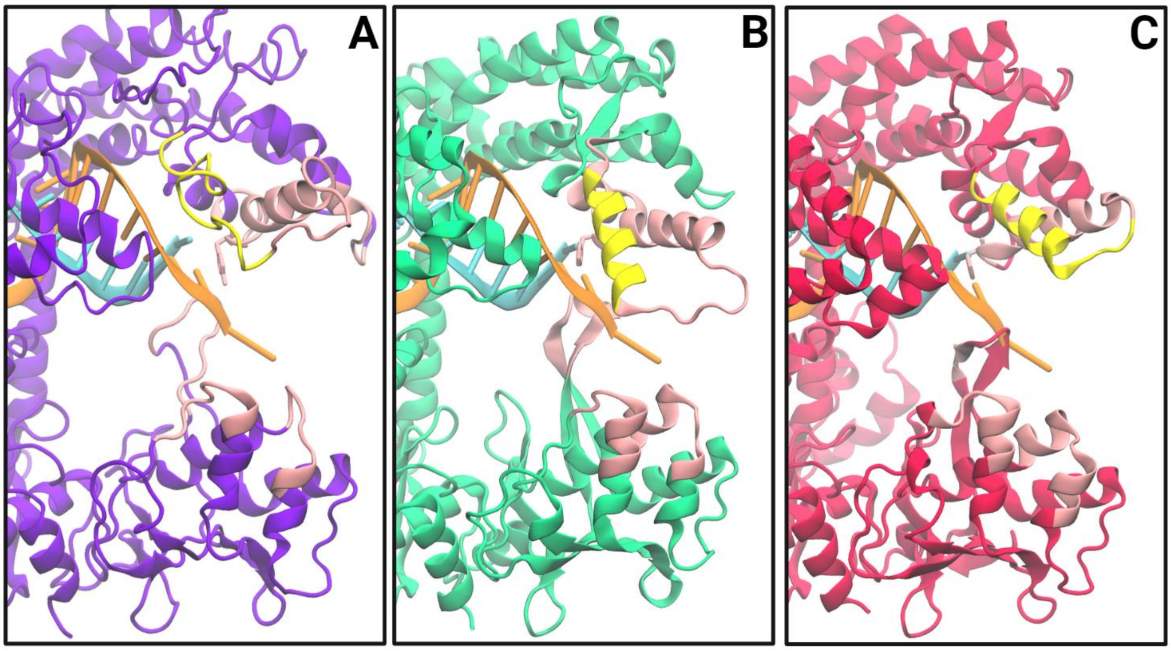

Figure S21: *Schematic of residues involved in REC2-NUC interactions (pink), and 1D DNA diffusion (yellow), overlaying (A) FnCas12a -6GTG, (B) LbCas12a -AF2 model, and (C) AsCas12a -AF2 model.*

Supplementary Tables

Cas12a	T (°C)	k_{NTS} (s^{-1})	k_{TS} (s^{-1})	DNA topology	Buffer
Lb[27]	25	0.203 ± 0.048	0.012 ± 0.004	(-) SC plasmid	10 mM Tris-HCl, pH 7.5, 100 mM NaCl, 10 mM $MgCl_2$, 0.1 mM DTT, 5 µg/ml BSA
Lb[26]	25	0.324 ± 0.0073	0.014 ± 0013	(-) SC plasmid	10 mM Tris-HCl, pH 7.5, 100 mM NaCl, 10 mM $MgCl_2$, 0.1 mM DTT, 5 µg/ml BSA
Lb*	30	0.199 ± 0.005	0.035 ± 0.026	(-) SC plasmid	10 mM Tris-HCl, pH 7.5, 50 mM NaCl, 10 mM $MgCl_2$, 5 µg/ml BSA, 0.1 mM DTT
As[20]	25	0.052 ± 0.6	0.0051 ± 0.2	Short linear	50 mM Na-MOPS, pH 7.0, 120 mM NaCl, 5 mM $MgCl_2$, 2 mM DTT
As*	30	0.133 ± 0.008	0.006 ± 0.002	(-) SC plasmid	10 mM Tris-HCl, pH 7.5, 50 mM NaCl, 10 mM $MgCl_2$, 5 µg/ml BSA, 0.1 mM DTT
As[25]	37	0.232 ± 0.059	0.061 ±0.010	Short linear	10 mM Tris-Cl, pH 7.9, 150 mM KCl, 5 mM $MgCl_2$, 1 mM TCEP
As[28]	37	0.029 ± 0.002	0.11 ± 0.023	Short linear	10 mM Tris-HCl, pH 8.0, 100 mM NaCl, 0.9 mM EDTA, 1 mM DTT, 5mM $MgCl_2$
As[28]	37	0.041 ± 0.012	0.19 ± 0.011	Short linear	10 mM Tris-HCl, pH 8.0, 100 mM NaCl, 0.9 mM EDTA, 1 mM DTT, 10mM $MgCl_2$
As[18]	37	0.10 ± 0.012	0.013 ± 0.0012	Short linear	50 mM HEPES, pH 7, 150 mM NaCl, 5 mM $MgCl_2$, 2 mM DTT, 0.2 mg/mL BSA
Fn*	30	0.561 ± 0.079	0.254 ± 0.034	(-) SC plasmid	10 mM Tris-HCl, pH 7.5, 50 mM NaCl, 10 mM $MgCl_2$, 5 µg/ml BSA, 0.1 mM DTT
Fn[14]	37	0.0075 ± 0.0012	0.0011 ± 0.00018	Short linear	20 mM Bicine-HCl pH 8, 150mM KCl, 0.5mM TCEP, 5 mM $MgCl_2$
Fn[23]	37	0.0483 ± 0.006	0.0338 ± 0.002	Short linear	20 mM HEPES, pH 7.5, 150 mM KCl, 5% glycerol, and 0.5 mM DTT, 5 mM $MgCl_2$

Table S1: *Mean rates of NTS and TS cleavage (with s.d.) from published literature. *Data from this study.*

	k_{NTS} (s^{-1})			k_{TS} (s^{-1})		
	Mean	S.D.	N	Mean	S.D.	N
FnWT	0.561	0.079	3	0.254	0.034	3
FnY410A	0.379	0.021	3	0.988	0.089	3
FnFLX	0.362	0.048	3	0.291	0.047	3
FnΔLoop	0.072	0.008	3	0.060	0.023	3
LbWT	0.199	0.005	3	0.049	0.005	3
LbW355A	0.080	0.013	3	0.163	0.010	3
LbFLX	0.070	0.017	3	0.114	0.031	3
LbΔLoop	0.204	0.030	3	0.066	0.007	3
AsWT	0.133	0.008	3	0.006	0.002	3
AsW382A	0.092	0.012	3	0.028	0.010	3
AsFLX	0.132	0.023	3	0.001	1.4e-4	3
AsΔLoop	0.083	0.008	3	0.002	8.0e-5	3
AsP1153A	0.103	0.024	3	0.006	4.6e-4	3
AsFLX-2	0.084	0.016	3	0.003	0.001	3

Table S2: *Mean rates of NTS and TS cleavage, with standard deviation and n.*

**Cas12a**	**Amino acids**	**Mutation**
FnFLX	D1158-D1164	GSGSGSG substitution (inclusive)
FnΔLoop	N1153-T1165	Deletion (inclusive)
LbFLX	K1079-D1085	GSGSGSG substitution (inclusive)
LbΔLoop	R1073-E1088	Deletion (inclusive)
AsFLX	E1165-F1169	GSGSG substitution (inclusive)
AsΔLoop	P1162-Y1173	Deletion (inclusive)
AsP1153A	P1153	Substitution to alanine
AsFLX-2	E1144-I1155	Truncation/substitution to GSG
**AsΔLoop-2*	*D1137-A1156*	*Deletion (inclusive)* ****no protein expression***

Table S3: *Mutations made to disrupt NUC loops.*

Orthologue	Domain	Residue #
FnCas12a	REC2	405-445; 463-469
	NUC loop	1154-1162
	NUC	1097-1104; 1196-1203
LbCas12a	REC2	345-394
	NUC loop	1076-1085
	NUC	1018-1027; 1117-1127
AsCas12a	REC2	382-414
	NUC loop	1169-1172
	NUC	1088-1096; 1202-1215

Table S4: *Protein contacts involved in REC2-NUC 'clamping', from MD simulation data.*

	**Sequence**	**T°(a)**
**Vector subcloning**		
fn_codingseq_f	AGATATACATATGAGCATCTACCAGGAG	61
fn_codingseq_r	TCTTCTTTGGGGCATAGTCGGGGACATC	61
as_codingseq_f	AGATATACATATGACACAGTTCGAGGGC	64
as_codingseq_r	GGGACATCATTTAGGCATAGTCGGGGAC	64
pet_fninsert_f	CGACTATGCCCCAAAGAAGAAGCGGAAG	59
pet_fninsert_r	AGATGCTCATATGTATATCTCCTTCTTAAAGTTAAAC	59
pet_asinsert_f	CTATGCCTAAATGATGTCCCCGACTATG	58
pet_asinsert_r	ACTGTGTCATATGTATATCTCCTTCTTAAAGTTAAAC	58
**Mutagenesis**		
fn_y410a_f	GTTTGATGACGCTTCCGTGATTGGGACC	66
fn_y410a_r	ACCTGCTGTGACAGGTCT	66
fn_e1006a_f	GTGGTGTTCGCGGATCTGAAC	58
fn_e1006a_r	AATGGCATTGTATTCGATG	58
fn_nucLOOPdel_f	AGGGAGGTGTACCCAACC	65
fn_nucLOOPdel_r	GATCAGGCGAGATCCGAAG	65
fn_flexstem_f	CGGGTCCGGGACTAGGGAGGTGTACCCA	62
fn_flexstem_r	GACCCGGACCCGGAATTTCGAAAGTTGATCAGG	62
as_w382a_r	AGGGCGCTGCTGATTGTC	68
as_w382a_f	GTGCGACCACGCGGATACACTGAGGAATG	68
as_e993a_f	GGTGGTGCTGGCCAACCTGAATTTC	64
as_e993a_r	ACGGCCTGGTAGTGGATC	64
as_p1153a_f	CAAGGGCACCGCGTTCATCGCCG	67
as_p1153_r	GCGTCAAACTGTGTCTCGTTC	67
as_flx2_f	TGGCGCCGGCAAGAGAATCGTG	65
as_flx2_r	GAGCCGTTCTTCTCGAACACGATATCCC	65

as_nucLOOPdel_f	CGGGACCTGTATCCTGCC	67
as_nucLOOPdel_r	CACGATTCTCTTGCCGGC	67
as_flexstem_f	CGGGTCCACCGGCAGATACCGGGAC	69
as_flexstem_r	GACCCGGAGATCACTGGCACGATTCTCTTGC	69
lb_w355a_f	CTTCGGCGAGGCGAACGTGATC	56
lb_w355a_r	ATATCCTTGGAGATTGTG	56
lb_e925a_f	ATCGCCCTGGCGGACCTGAAC	64
lb_e925a_r	CACGGCATCGTACTTCTCCAC	64
lb_nucLOOPdel_f	GTGTGCCTGACCAGCGC	69
lb_nucLOOPdel_r	GATCCGGTTGCCGTAGGAG	69
lb_flxstem_f	CGGGTCCGGGTGGGAGGAGGTGTGCCTG	68
lb_flxstem_r	GACCCGGACCCAGGATTCCGGAAGATTCTGATCCGG	68

Table S5: Sequences of cloning primers.

Name	Sequence
Mini_locus_top	ATAAGGAGATATACCATGGGAATTTCTACTGTTGTAGATTATGGGTATAAATGGGCTCGCGAAATTTCTACTGTTGTAGATTATGGGTATAAATGGGCTCGCGAAATTTCTACTGTTGTAGATGAATTCGAGCTCGGCGCGCC
Mini_locus_bottom	GGCGCGCCGAGCTCGAATTCATCTACAACAGTAGAAATTTCGCGAGCCCATTTATACCCATAATCTACAACAGTAGAAATTTCGCGAGCCCATTTATACCCATAATCTACAACAGTAGAAATTCCCATGGTATATCTCCTTAT
FQ ssDNA	5'-/56-FAM/TTT TTT TTT/ZEN/TTT/3IaBkFQ/ -3'
TS	CCCGGTGTCACGCCACTTGACAGGCGAGTAACAGACATGGACCATCAGGAAACATTAACGTACTGATGTTAACAGCTGACCCAATAAGTGGCAGAG
TS_truncated	TAACAGACATGGACCATCAGGAAACATTAACGTACTGATGTTAACAGCTGACCCAATAAGTGGCAGAG

Table S6: Oligonucleotide sequences used in ‘locus’ cloning and *trans* cleavage assays.

**crRNAs for in vitro cis and trans cleavage assays**	
Lb_23	UAAUUUCUACUAAGUGUAGAUCUGAUGGUCCAUGUCUGUUACUC
Lb_20	UAAUUUCUACUAAGUGUAGAUCUGAUGGUCCAUGUCUGUUA
As_23	UAAUUUCUACUCUUGUAGAUCUGAUGGUCCAUGUCUGUUACUC
As_20	UAAUUUCUACUCUUGUAGAUCUGAUGGUCCAUGUCUGUUA
Fn_23	UAAUUUCUACUGUUGUAGAUCUGAUGGUCCAUGUCUGUUACUC

Fn_20	UAAUUUCUACUGUUGUAGAUCUGAUGGUCCAUGUCUGUUA
**crRNAs for human cell gene editing via RNP electroporation**	
Lb_DNMT1-3	UAAUUUCUACUAAGUGUAGAUCUGAUGGUCCAUGUCUGUUACUCG
Lb_DNMT1-7	UAAUUUCUACUAAGUGUAGAUGCUCAGCAGGCACCUGCCUCAGCU
Lb_AGBL1	UAAUUUCUACUAAGUGUAGAUGAUUGAAGGAAAAGUUACAAAGGU
As_DNMT1-3	UAAUUUCUACUCUUGUAGAUCUGAUGGUCCAUGUCUGUUACUCG
As_DNMT1-7	UAAUUUCUACUCUUGUAGAUGCUCAGCAGGCACCUGCCUCAGCU
As_AGBL1	UAAUUUCUACUCUUGUAGAUGAUUGAAGGAAAAGUUACAAAGGU
Fn_DNMT1-3	UAAUUUCUACUGUUGUAGAUCUGAUGGUCCAUGUCUGUUACUCG
Fn_DNMT1-7	UAAUUUCUACUGUUGUAGAUGCUCAGCAGGCACCUGCCUCAGCU
Fn_AGBL1	UAAUUUCUACUGUUGUAGAUGAUUGAAGGAAAAGUUACAAAGGU

Table S7: RNA oligonucleotide sequences.

Site name	site sequence	primer set name*	Forward primer	Reverse primer
dnmt1-3	TTTCCTGATGGTCCATGTCTGTTA	DNMT1-3 / TTTC-2	TCCCTTAGCACTCTGCCACTTAT	GTTAAAAACACAACATCAGTGCATGT
dnmt1-7	TTTGGCTCAGCAGGCACCTGCCTC	DNMT1-7 /TTTG-2	AGCAGGCCTTTGGTCAGGTT	AGACATGGACCATCAGGAAACAT
agbl1	TTTAGATTGAAGGAAAAGTTACAA	AGBL1 /TTTA-6	GAAGAGAAATCTGCGTGGAGAGA	GAAAACCCCCAAAAATCCCA
dnmt1-3-OT1	TTTCCTGATGGTCCATGTCTGAAT	WT-AsCas12a-TTTC-2-chr1	CCTATTCTTCCGCCATTTTCC	CTGAATGCCTGCTATGTACACACA
dnmt1-3-	TTTCCTGATGGTCCACATCTGTTA	enAsCas12a-TTTC-	TGCCACATTACCCTCTAAAAGTCA	TGCGCAGTGTGTTTAGGAAGTT

OT 2		2-chr11		
dnmt1-3-OT3	TTTCCTGATGGTCCATATCTGTGG	enAsCas12a-TTTC-2-chr10	TCAGGTGATGGTTTGGCAATC	ATAGAAAGCCTCCCCACCTAAGG
dnmt1-3-OT4	TTTCCTGATGGTCCATACCTGTTA	enAsCas12a-TTTC-2-chrX	CTCTTCCCCTCAACCACTAAATATG	TCTGAGGTTTTCTTCCCTTTTCC
dnmt1-7-OT1	AGCAGCTCAGCAGGCACCTGCCTT	enAsCas12a-TTTG-2-chr21	CAGCCAGGGCCTCATTAAAC	CATGTTGAATGTTGGCACGAA
dnmt1-7-OT2	AACAGCTCAGCAGACACCTGCCAA	enAsCas12a-TTTG-2-chr2	TTGCCATGACAACAACTCAGG	GGACGCAATGTCAGCTGTGT
dnmt1-7-OT3	TTTAGCTCAGCTGACACCTGCCCA	enAsCas12a-TTTG-2-chr12	TGTTGCATAGGCTTGTTCTGTTC	TTGTTAGGGTTCATTGTAGCTGACA
abgl1-OT1	TTTAGATTAATGGAAAAGTTACAA	enAsCas12a-TTTA-6-chr6	CACTGCACCCGGCCATA	TGTGGAGTCTTGAACTGGATCTTG
abgl1-OT2	TTTAGACTTAAAGAAAAGTTACAA	enAsCas12a-TTTA-6-chr8	GTGGTAGTACTTCCTTCATACTGAGCA	GTCCCGTGGAGTAGTTGATGTG
abgl1-OT3	CTTGGATTAAAGGAAAAGCTACAA	enAsCas12a-TTTA-6-chr10	TGGAAATAAGCAATCAGGCCTT	TTTGTTTTGTCTTTCCTTACATCGTT

Table S8: Primers for high-throughput sequencing. *Sites names as per Kleinstiver 2019

"Kleinstiver, B.P., Sousa, A.A., Walton, R.T. et al. Engineered CRISPR–Cas12a variants with increased activities and improved targeting ranges for gene, epigenetic and base editing. Nat Biotechnol 37, 276–282 (2019). https://doi.org/10.1038/s41587-018-0011-0"
